# Natural product Erianin: mitigating FOLFOX toxicity and enhancing against colorectal cancer

**DOI:** 10.3389/fchem.2025.1650197

**Published:** 2025-08-22

**Authors:** Fuqin Yang, Zeqiao Li, Haishan Zhang, Mingxiao Zhang, Zhenwei He, Xiaoqin Zheng, Yingru Zhu, Pei Long, Ruirui Ding, Zhengbin Lin, Lijuan Deng

**Affiliations:** ^1^ Guangzhou Key Laboratory of Formula-Pattern Research Center, School of Traditional Chinese Medicine, The Fifth Affiliated Hospital of Jinan University (Heyuan Shenhe People’s Hospital), Jinan University, Guangzhou, China; ^2^ State Key Laboratory of Bioactive Molecules and Druggability Assessment, Guangdong Basic Research Center of Excellence for Natural Bioactive Molecules and Discovery of Innovative Drugs, College of Pharmacy, Jinan University, Guangzhou, China; ^3^ Xinhua Hospital Affiliated to Shanghai Jiao Tong University School of Medicine, Changxing Branch, Shanghai, China; ^4^ Guangdong Provincial Key Laboratory of Traditional Chinese Medicine Informatization, Jinan University, Guangzhou, China

**Keywords:** Erianin, FOLFOX regimen, colorectal cancer, toxicity reduction, efficacy enhancement

## Abstract

**Introduction:**

Colorectal cancer (CRC) is a prevalent malignant tumor of the digestive tract. The FOLFOX regimen (oxaliplatin + calcium folinate + 5-fluorouracil) serves as the primary treatment for advanced CRC clinically, yet its application is significantly limited by substantial toxic side effects. Erianin, a natural compound from Chinese medicine *Dendrobium chrysotoxum* Lindl, demonstrates significant potential in both tumor growth inhibition and chemotherapy toxicity reduction. This study aims to investigate the potential of Erianin in reducing the toxicity of the FOLFOX regimen while enhancing its antitumor efficacy.

**Methods:**

This study integrated network toxicology and molecular docking to predict the potential targets of Erianin in alleviating FOLFOX-induced side effects. Using an orthotopic MC38 CRC transplantation model, we conducted a comprehensive evaluation of the effects of Erianin in mitigating FOLFOX-induced changes in body weight changes, hematological parameters, and histopathology of major organs (heart, liver, spleen, kidneys, and intestines). IHC analysis elucidated alterations in intestinal barrier proteins, AKT1/mTOR pathway, and tumor proliferation and apoptosis biomarkers. Tumor progression was dynamically monitored by *in vivo* imaging.

**Results:**

The results showed that Erianin improved weight loss, pathological changes in organs, and reduction in peripheral blood cell counts (WBC, RBC, HGB, PLT, HCT) caused by FOLFOX in mice. Erianin reversed the inhibition of intestinal tight junction proteins (e.g. ZO-1, Occludin, Claudin-5) and AKT1/mTOR pathway caused by FOLFOX. In addition, the tumor size was significantly reduced in the combination group, and the expression of the apoptosis marker Cleaved Caspase-3 was up-regulated, and the proliferation markers Ki67/PCNA were down-regulated.

**Discussion:**

Erianin can enhance the anti-CRC effect of FOLFOX, and mitigates FOLFOX-induced toxicity by activating the AKT1/mTOR pathway.

## 1 Introduction

Colorectal cancer (CRC) ranks as the third most common cancer globally, exceeded only by lung and breast cancer. Since the 21st century, the global incidence of CRC has continued to rise. The burden of CRC is projected to increase to 3.2 million new cases and 1.6 million deaths by 2040, posing a serious threat to public health (Morgan et al., 2023). Chemotherapy remains the cornerstone of treatment for patients in the middle to advanced stages. Currently, the FOLFOX regimen (oxaliplatin + calcium folinate + 5-fluorouracil), as a first-line clinical treatment, significantly improves patient survival rate. However, its associated toxic side effects severely limit its clinical application. Statistically, over 70% of patients develop oxaliplatin-induced peripheral neuropathy (Marcotti et al., 2023). About 30% of patients experience myelosuppression during 5-fluorouracil treatment (Dekker et al., 2019; Kogo et al., 2011). Notably, gastrointestinal toxicity is a prominent adverse effect of chemotherapy, with mucositis occurring in approximately 40%–80% of patients receiving chemotherapy, and nearly one-third of patients on 5-fluorouracil or irinotecan progressing to grade 3–4 severe diarrhoea (Stein et al., 2010; Thomsen and Vitetta, 2018). Of these, intestinal barrier dysfunction is particularly critical, as it is not only a direct trigger for dose-limiting diarrhoea and chemotherapy interruption, but may also further worsen patient survival outcomes by disrupting intestinal mucosal integrity (Calvo et al., 2002). Although existing adjuvant drugs (e.g., Dolasetron and Ranitidine) partially alleviate specific toxicities, their efficacy volatility and additional side effects limit clinical application (Gor et al., 2023; Nielsen et al., 2002). Therefore, there is an urgent need to develop novel synergistic agents with high efficacy and low toxicity.

According to Traditional Chinese Medical (TCM) theory, the ancient practice of utilizing food as medicine is conceptualized as “medicine-food homology” (*yào shí tóng yuán*) - a doctrine deeply rooted in Chinese cultural heritage that emphasizes health preservation and adjunctive therapy through dietary regulation (Zhang et al., 2025). It has gained significant attention in anti-tumor drug development. Their safety profile, low toxicity and multi-target regulatory properties make them promising candidates for tumor-adjuvant therapies (Li et al., 2025). These substances can be consumed for long-term to prevent disease onset or progression while serving as complementary treatments to improve patients’ quality of life. Notably, their anti-tumor potential offers substantial promise for cancer management. Although their efficacy may be less pronounced than first-line clinical drugs, their minimal toxicity enables prolonged administration, providing essential nutrients and reducing treatment-related discomforts. This dual action demonstrates a unique advantage in anti-tumor treatment: enhancing efficacy while reducing toxicity. Currently, research on applying medicinal foods in tumor treatment remains limited (Li M. et al., 2024). Therefore, further basic and clinical investigations are warranted to explore their full potential applications in oncology.

*Dendrobium chrysotoxum* Lindl, a traditional dual-purpose medicinal food plant in China, was officially included in China’s medicinal food catalog in 2023. Traditionally consumed fresh, in soups, cooked dishes, juices, teas, and wines. Its long-standing culinary use with no reported adverse reactions underscores its safety profile (Zhang et al., 2023). Erianin, an important bioactive constituent of *D. chrysotoxum* Lindl, exhibits significant anti-tumor and anti-inflammatory properties. Studies have shown that Erianin not only directly induce apoptosis in multiple tumor cell lines but also regulates epithelial-mesenchymal transition (EMT) to inhibit tumor cell invasion and migration (Li R. et al., 2024). In contrast to the established protective effects of curcumin through Axl/GSK-3β/NF-κB pathway modulation, its mechanisms against chemotherapy-induced toxicity remain incompletely characterized, particularly for intestinal damage where only basic apoptotic markers have been examined (Cai et al., 2022; Moutabian et al., 2022). Resveratrol also primarily ameliorates chemotherapy-induced cardiotoxicity and nephrotoxicity (Xiao et al., 2019). The current study bridges this critical knowledge gap by employing an innovative integration of network toxicology and molecular docking to systematically investigate Erianin’s protective actions against FOLFOX regimen toxicity. Our findings demonstrate that Erianin not only preserves intestinal barrier integrity through tight junction protein regulation but also orchestrates a comprehensive cytoprotective response via AKT1/mTOR pathway modulation. This multi-targeted approach, validated through both computational predictions and experimental model, reveals Erianin’s unique potential as a novel chemoprotective adjuvant capable of addressing the complex toxicity profile of FOLFOX chemotherapy while maintaining therapeutic efficacy.

## 2 Materials and methods

### 2.1 Reagents

Erianin (Cat. # HE258917, purity = 99.311% verified by HPLC) was obtained from Herbest (Baoji, China). The detailed HPLC methodology and analytical parameters for Erianin purification and quality control have been comprehensively documented in the Supplementary Material. Fluorouracil (Cat. # GC14466) and Oxaliplatin (Cat. # GC17716) were obtained from GlpBio Technology (Montclair, USA). Calcium folinate (Cat. #F7878) was purchased from Sigma-Aldrich (St Louis, USA). Fetal bovine serum (FBS, Cat. #A5670801), penicillin and streptomycin (PS, Cat. #15140122), and Dulbecco’s modified Eagle’s medium (DMEM, Cat. # 11995065) were purchased from Gibco (Grand Island, USA). Matrigel (Cat. #354230) was obtained from Corning (Corning, NY, AZ, USA). D-Luciferin, Sodium Salt (Cat. # 40901ES03) was purchased from Yeasen Biotechnology (Shanghai, China). Hematoxylin and eosin (H&E, Cat. #C0105S) staining kit was purchased from Beyotime (Shanghai, China).

### 2.2 Cell lines and cell culture

Mouse MC38 cells were purchased from the Cell Bank of the Chinese Academy of Sciences (Shanghai, China). Cells were cultured in high-glucose DMEM medium containing 10% FBS, 1% PS solution. The cells were placed in a 37 °C constant temperature incubator containing 5% CO_2_, and the cells in logarithmic growth phase were taken for subsequent experiments.

### 2.3 Network toxicology analysis

Target genes associated with FOLFOX were identified using the Comparative Toxicogenomics Database (CTD; https://ctdbase.org/). Targets related to intestinal injury were screened utilizing the GeneCards database (https://www.genecards.org/) and the Online Mendelian Inheritance in Man (OMIM) database (https://www.omim.org/). Targets linked to both FOLFOX toxicity and intestinal injury were intersected using Venny 2.1.0 website (https://bioinfogp.cnb.csic.es/tools/venny/) to obtain common targets. Potential targets of Erianin were collated from the CNKI (https://c61.oversea.cnki.net/) and PubMed (https://pubmed.ncbi.nlm.nih.gov/) platforms. Additionally, the chemical structural of Erianin was submitted to the SwissTargetPrediction database (http://swisstargetprediction.ch/) to computationally predict its molecular targets. The intersecting targets between Erianin and FOLFOX-induced intestinal injury were obtained using Venny 2.1.0. The String (https://string-db.org) platform was used to construct protein-protein-interaction (PPI) networks. Key signaling pathways were subsequently analyzed using Gene Ontology (GO) enrichment and Kyoto Encyclopedia of Genes and Genomes (KEGG) pathway analysis.

### 2.4 Molecular docking

The molecular docking between Erianin and the key target AKT1 was performed, and the 3D structures of AKT1 (7WM2) was downloaded using the PDB (http://www.rcsb.org/) database. PubChem (https://pubchem.ncbi.nlm.nih.gov/) was utilized to find the chemical conformational information of Erianin. Receptor and ligand pre-processing, such as removal of water molecules and supplementation of hydrogen ions, was carried out using AutoDock Vina 1.5.7 software, followed by saving as a pdbqt file for molecular docking, and finally, the docking results were visualized and analyzed using PyMol 2.4.0.

### 2.5 Animals

15 SPF-grade male C57BL/6 mice (8 weeks old) were purchased from Zhuhai BesTest Bio-Tech Co., Ltd. and raised at Jinan University Laboratory Animal Management Center. Mice were group-housed (n = 5 per cage) in a barrier-maintained facility. The bedding, drinking water bottle and other items used by the mice were sterilized by high temperature and brought into the barrier facility. The feed of the mice was irradiated and maintained by vacuum packaging, and the water consumed by the mice was purified. All animal experimental operations in this study followed the Guidelines for the Animal Experimental Ethics Committee of Jinan University (IACUC-20240428-06).

### 2.6 The orthotopic MC38 CRC model and *in vivo* experiments

MC38 cells in logarithmic growth phase were harvested. The cell pellet was resuspended in pre-cooled 1× PBS and mixed with Matrigel at a 2:1 ratio (PBS: Matrigel). Finally, the cell density was adjusted to 1 × 10^7^ cells/mL. 100 μL cell suspension was inoculated onto the cecal wall. Tumor-bearing mice were then randomized into three groups (n = 5/group): vehicle group, FOLFOX group, and Erianin + FOLFOX combination group. The FOLFOX group: Single intraperitoneal (i.p.) dose of FOLFOX regimen (5-fluorouracil 50 mg kg^-1^, oxaliplatin 10 mg kg^-1^, calcium folinate 90 mg kg^-1^); the combination group: Erianin (10 mg kg^-1^) pretreatment followed by identical FOLFOX regimen (both i.p.); vehicle group: Equivalent volume of saline (i.p.). All non- FOLFOX groups received daily treatments for seven consecutive days. At designated time points, mice received intraperitoneal injections of D-luciferin (150 mg kg^-1^). After 10 min, animals were anesthetized with 1% pentobarbital sodium and imaged using the IVIS Lumina LT system (PerkinElmer, Waltham, MA, USA), standardized exposure times (30–60 s depending on signal intensity), and the quantitative region-of-interest (ROI) analysis methodology (statistical fluorescence intensity measurement by threshold-based background segmentation of fluorescent regions using Living Image software). After 7 days of treatment, mice showing a 15%–20% reduction in body weight were euthanized via cervical dislocation following anesthesia with pentobarbital sodium (50 mg·kg^-1^). Death was confirmed by observing cessation of breathing, heart rate, and absence of a pain response upon toe pressure. The final tumor weight was maintained below 10% of total body weight in all experimental groups, complying with institutional animal welfare guidelines and ethical requirements for humane endpoints.

### 2.7 Observe the appearance of the organ and calculate the organ index

Mouse spleen, liver and intestinal tissues were collected, rinsed with normal saline to remove blood, gently blotted dry on filter paper and weighed. Tissues were grouped and photographed for documentation. Organ indices were subsequently calculated.

### 2.8 Complete blood count

Following humane euthanasia, mouse peripheral blood was collected via retro-orbital puncture into pre-chilled 1.5 mL EP tubes containing EDTA anticoagulant. Tubes were immediately inverted gently for mixing. Blood samples were transported on ice to the inspection department of Jinan University Experimental Animal Management Center and analyzed within 30 min.

### 2.9 Hematoxylin and eosin stain (H&E) staining

The liver, spleen, heart, kidney and intestine of mice were extracted and fixed with 4% paraformaldehyde for more than 24 h. The tissue was placed in an embedded box and soaked in a cylinder containing 70%, 80%, 90%, and 95% gradient ethanol for 30 min, soaked in 100% ethanol I and II for 1 h, and soaked in xylene I and II for 30 min. Soak the wax with low melting point for 1 h and the wax with high melting point for 2 h. Embed using a tissue embedder and cut into 4 μm slices per slice. Then, the sheets were baked in an oven at 60 °C for 30 min, and soaked in xylene I and II for 15 min, and each of 100% ethanol, 95% ethanol, 90% ethanol, 85% ethanol, 80% ethanol, and 75% ethanol for 5 min. Then the sections were stained with hematoxylin for 2 min. If the tissue color was too dark, differentiation was performed. After differentiation, the tissue was changed to blue and eosin stained for 2 min. During this period, rinsed with running water. Finally, the gradient ethanol dehydration was carried out, neutral gum sealing was taken by the Olympus BX53 inverted epifluorescence microscope (Tokyo, Japan) to observe.

### 2.10 Immunohistochemistry (IHC)

Dehydration-embedded sections were processed following the same protocol as used for H&E staining, as previously described (Qi et al., 2022). The tissue sections were then subjected to antigen retrieval, membrane permeation, blocking. Primary antibodies included: Ki67 (Servicebio, #GB11141, 1:300), ZO-1 (Servicebio, #GB115686,1:300), Occludin (Servicebio, #GB11149, 1:300), Claudin 5 (Servicebio, #GB1190,1:300), Cleaved Caspase-3 (CST, #9661T, 1:400), PCNA (CST, #2586, 1:400), AKT1 (CST, #75692, 1:400), p-AKT1 (abcam, #ab81283, 1:400), mTOR (CST, #2983, 1:400), p-mTOR (CST, #2976, 1:400). The sections were incubated with HRP-labeled secondary antibody (Servicebio, GB23303, GB23301, 1:400) the next day and color development was performed using the DAB kit (Servicebio, #G1212, 1:50) for 1–5 min and hematoxylin counterstaining for 2 min. Images were captured using an Olympus BX53 inverted epifluorescence microscope.

### 2.11 Statistical analysis

GraphPad Prism 9.5.0 software was used for processing, and the data results were expressed by means ± SEM. Statistical differences between two groups were evaluated using a two-tailed unpaired *t*-test, and differences between more than two groups were evaluated using one-way ANOVA followed by Tukey’s *post hoc* test. When *p < 0.05*, the difference was considered statistically significant.

## 3 Results

### 3.1 Network toxicology analysis of FOLFOX-induced intestinal injury

**FIGURE 1:**
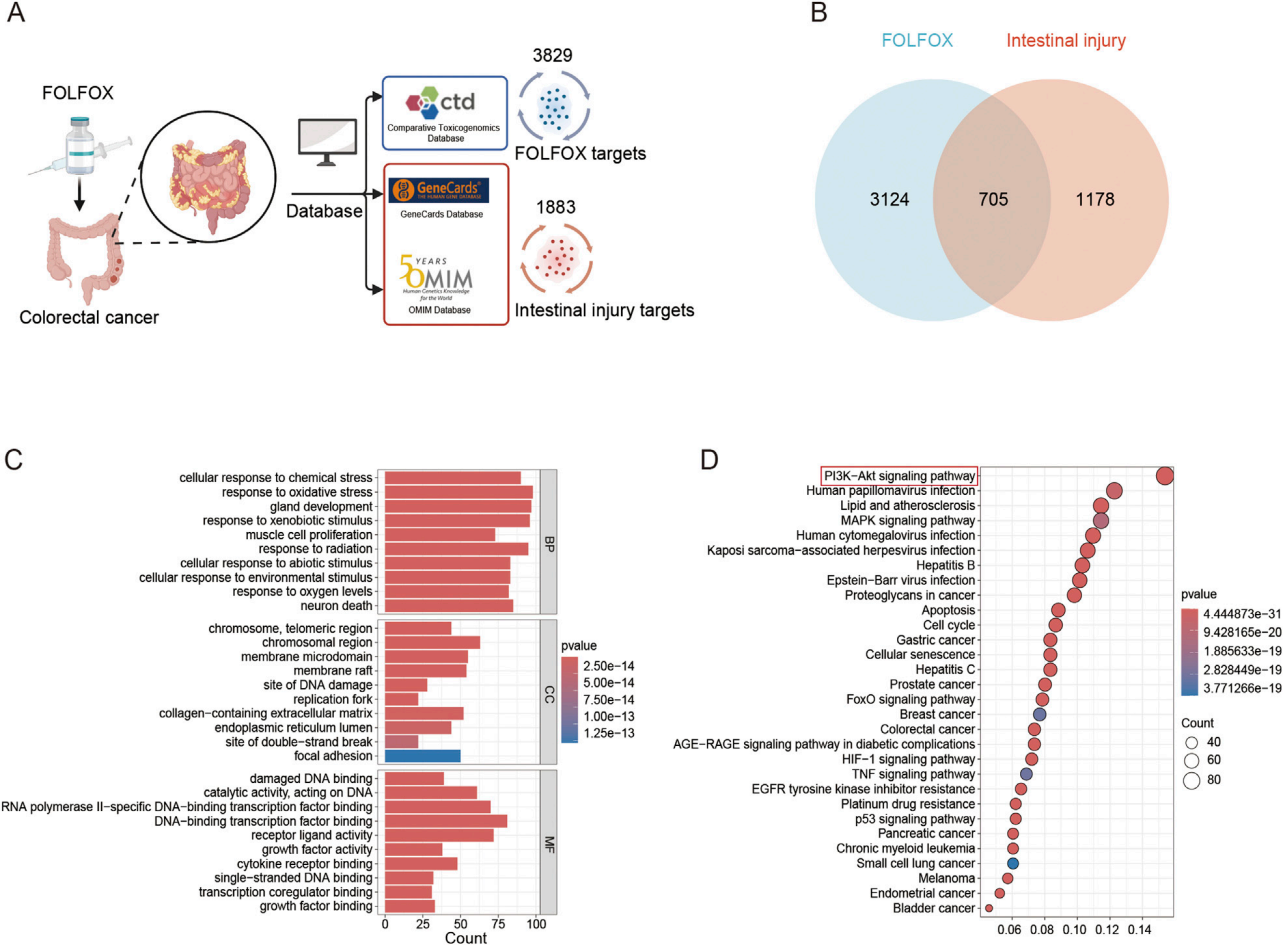
Network toxicology analysis. **(A)** Prediction of FOLFOX toxicity and intestinal injury targets using CTD, GeneCards, and OMIM databases. **(B)** Venn diagram of overlapping targets associated with FOLFOX toxicity and intestinal injury. **(C)** GO enrichment analysis across biological processes (BP), cellular components (CC), molecular functions (MF). **(D)** KEGG pathway enrichment.

Utilizing the CTD database, 3,829 FOLFOX-associated target genes were identified. Concurrently, 1,883 intestinal injury-related targets were retrieved from the GeneCards and OMIM databases after duplicate removal (Figure 1A). Venny 2.1.0 analysis revealed 705 intersection targets (Figure 1B). GO enrichment analysis mainly involves the stress response of cells to external stimuli, cell structural function and biomolecular interactions. Cellular stress responses include responses to changes in chemicals, oxidative pressure, radiation, exogenous substances and oxygen concentrations, including DNA repair, neuronal death and cell proliferation regulation. Structures such as chromosomal telomeres, membrane microdomains, and replication forks are involved in genomic stability and signaling. Molecular functions focus on DNA/RNA binding, transcriptional regulation, growth factor and receptor interaction, affecting cell proliferation, differentiation and environmental adaptation (Figure 1C). KEGG enrichment analysis mainly involves cancer, viral infection, metabolic diseases and cell regulation mechanisms. Among them, PI3K-AKT, MAPK, p53 and other signaling pathways are related to cell proliferation, apoptosis, cycle, and drug resistance. Pathways closely related to viral infection, such as HPV, HBV, EBV, etc., mainly promote tumorigenesis by hijacking the host. Metabolic-related pathways such as AGE-RAGE and HIF-1 are involved in complications of diabetes and hypoxia. In addition, inflammatory pathways such as TNF are involved (Figure 1D). These pathways reveal potential molecular mechanisms of FOLFOX-induced intestinal damage.

### 3.2 Erianin alleviates FOLFOX-induced intestinal toxicity with molecular docking predictions

**FIGURE 2:**
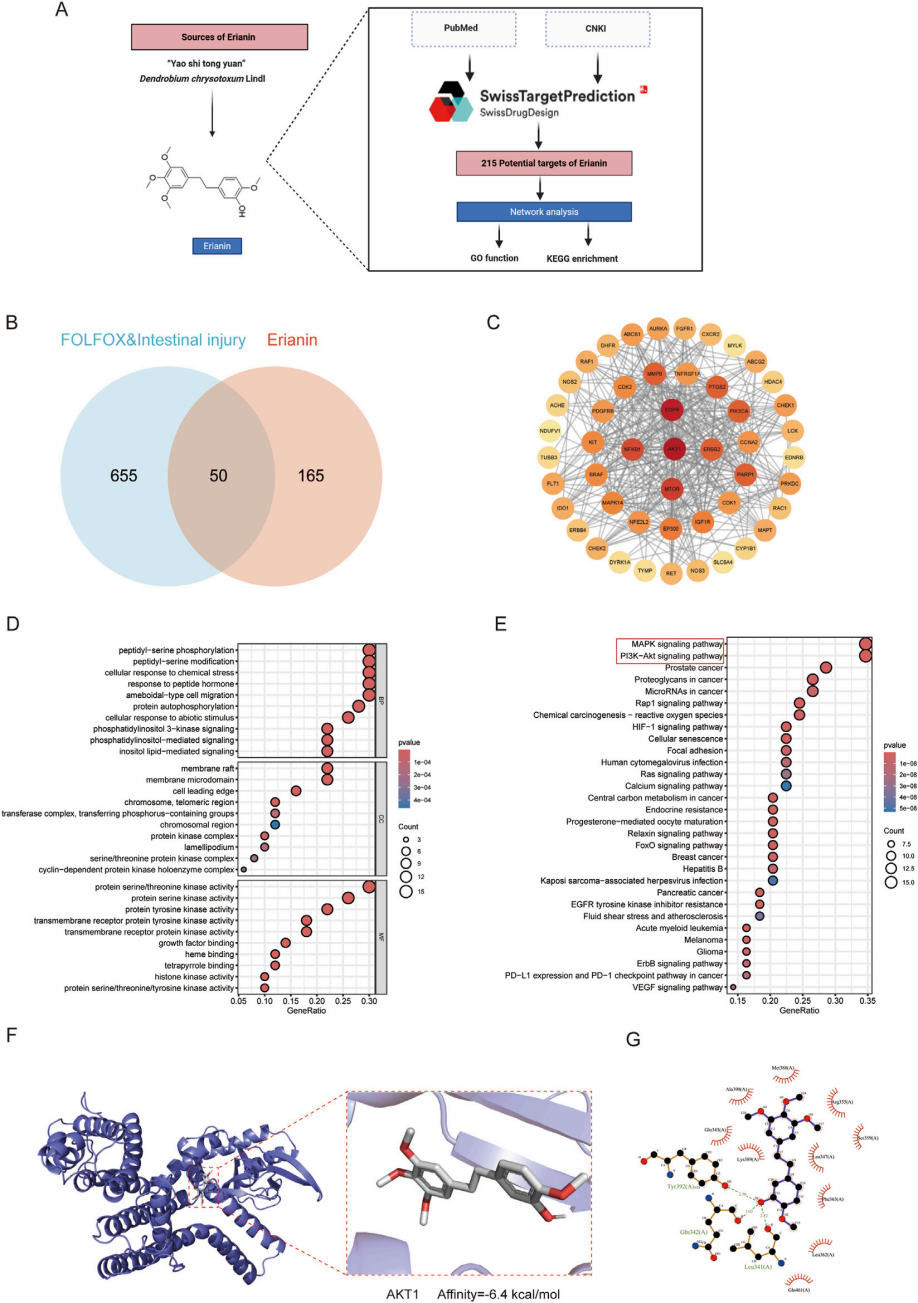
Potential mechanisms by which Erianin alleviates intestinal toxicity induced by FOLFOX regimen. **(A)** Technical pipeline for screening and predicting Erianin’s molecular targets. **(B)** Venn diagram analysis of shared targets between Erianin and FOLFOX-induced intestinal toxicity. **(C)** Protein-protein interaction network of shared targets between Erianin and FOLFOX-associated gut toxicity. **(D)** GO function analysis for BP, CC, MF. **(E)** KEGG enrichment analysis. **(F,G)** Visualization of Erianin-AKT1 binding site from molecular docking (Left: 3D binding conformation; Right: 2D interaction pattern).

Integration of literature mining and SwissTargetPrediction database analysis identified 215 potential therapeutic targets of Erianin (Figure 2A). Venny intersection analysis identified 50 overlapping targets between Erianin and FOLFOX chemotherapy-induced intestinal injury (Figure 2B). Using the STRING database, a PPI network was constructed with 50 nodes and 377 edges. Darker-colored proteins in the network were suggested to play more important roles (Figure 2C). GO enrichment analysis revealed predominant involvement in cellular signal transduction, metabolic regulation and synaptic activity (Figure 2D). The results of KEGG pathway analyses suggested that Erianin may alleviate FOLFOX chemotherapy-induced intestinal toxicity through multi-target synergistic effects. Significantly enriched, PI3K-AKT and MAPK signalling pathways, oxidative stress-related pathways (chemoattenuating-ROS, HIF-1), and so on. In addition, the enrichment of Rap1 and Focal adhesion pathway implied that Erianin may enhance the expression of intestinal epithelial tight junction proteins and maintain intestinal barrier integrity (Figure 2E). These findings provide a theoretical basis for the development of Erianin as an adjuvant to FOLFOX chemotherapy. Molecular docking analysis was performed to assess the interactions between Erianin and the top core target AKT1 (Figures 2F,G). Docking results showed that Erianin binds to AKT1 binding affinities of −6.4 kcal/mol. Erianin forms three predicted hydrogen bonds with AKT1 through residues Tyr-392, Gln-342 and Leu-341. Collectively, these data indicating that the interaction with key target AKT1 may underlie Erianin’s protective effects against chemotherapy-induced toxicity.

### 3.3 Erianin mitigates FOLFOX-induced poor condition and weight loss

**FIGURE 3:**
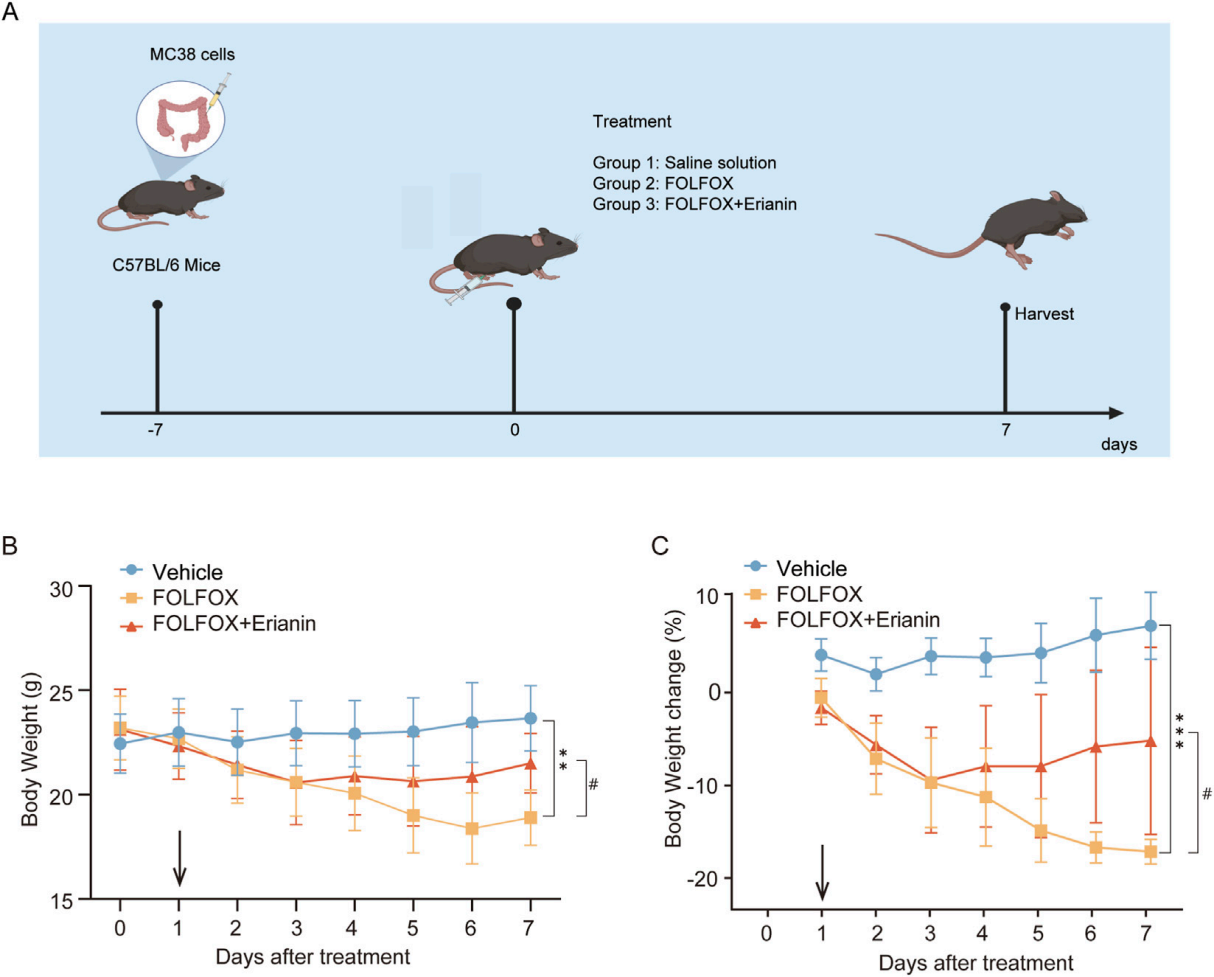
Erianin ameliorates FOLFOX-induced weight loss in mice. **(A)** Schematic diagram of the mouse dosing regimen. **(B)** The weight of the mice (n = 5). **(C)** Results of weight change in mice (n = 5). Data were presented as mean ± SEM. **p < 0.01, ***p < 0.001 vs. vehicle; ^*#*^ *p < 0.05* vs. FOLFOX monotherapy.

To validate the ability of Erianin to alleviate FOLFOX-induced toxicity and side effects *in vivo*, we established an orthotopic colorectal cancer xenograft model in C57 mice. The schematic diagram of the mouse dosing regimen is shown in Figure 3A. Daily monitoring revealed that Erianin co-administration attenuated FOLFOX-associated weight loss and improved the status of mice during the treatment period. Specifically, the mice in the FOLFOX monotherapy group exhibited severe toxicity: dull unkempt fur, localized alopecia, reduced responsiveness, and hunched posture. The mice in the Erianin + FOLFOX combination group demonstrated significantly improved the movement coordination and response sensitivity versus monotherapy. Notably, the combination therapy group showed slower rate of weight reduction, higher terminal body weight at endpoint (Figure 3B), and reduced percentage weight loss (Figure 3C). These results suggest that Erianin’s protective role against FOLFOX-induced weight loss.

### 3.4 Erianin ameliorates FOLFOX-induced organ abnormal appearance

**FIGURE 4:**
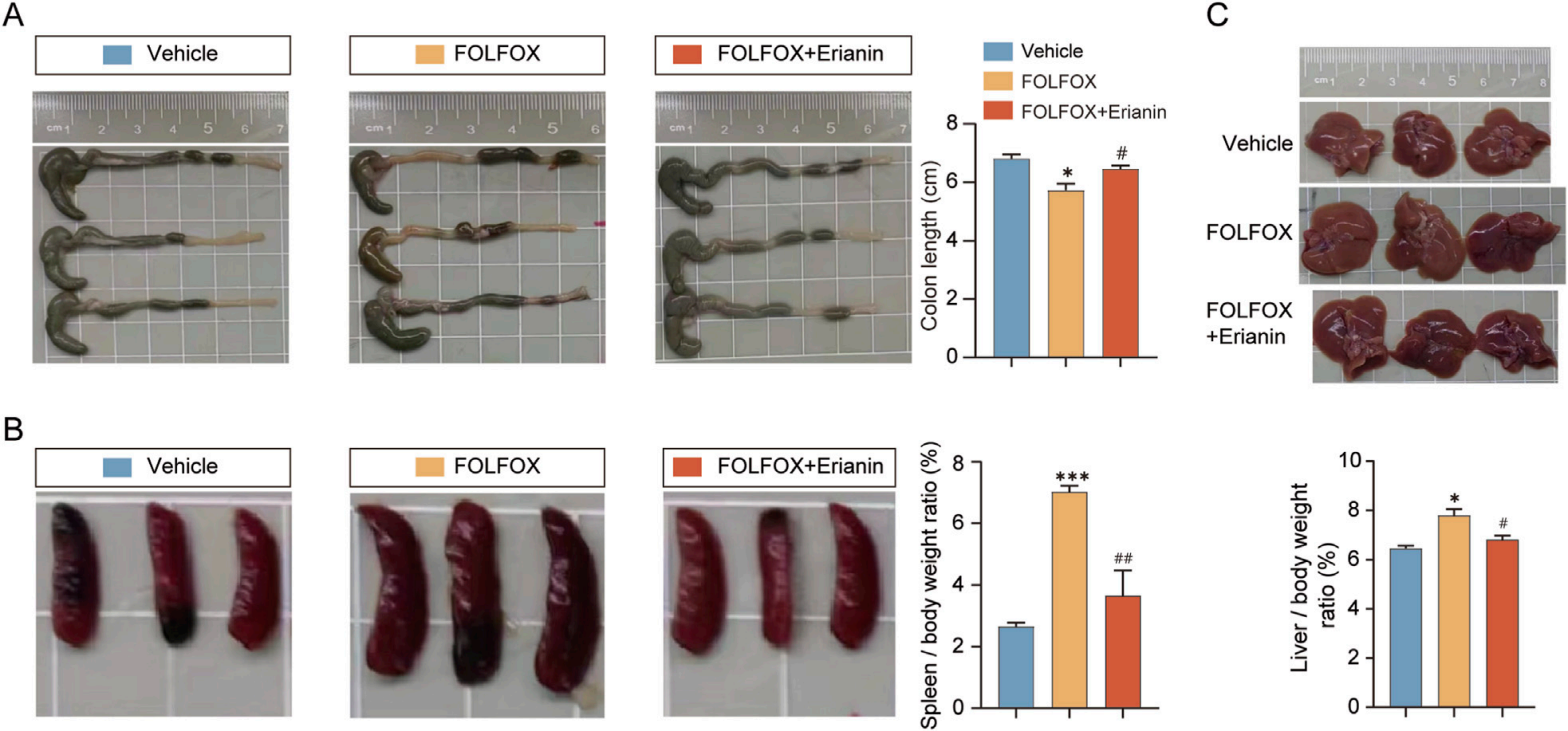
Erianin improves the abnormal appearance of mouse organs caused by FOLFOX. **(A)** Representative colon images (Left) and length statistics (Right) across groups. **(B)** Appearance of mouse spleens (Left) and quantitative analysis (Right). **(C)** Images of mouse livers (upper) and quantitative analysis (lower). n = 5. Data were presented as mean ± SEM. *p < 0.05, ***p < 0.001 vs. vehicle; ^*#*^ *p < 0.05,* ^*##*^ *p < 0.01* vs. FOLFOX monotherapy.

The major organs, such as colon, spleen and liver appearance and morphology of mice in each group were observed. Compared with the mice in the vehicle group, the colon length of the mice in the FOLFOX group was significantly shortened, and the Erianin + FOLFOX combination group reversed this phenomenon (Figure 4A). In addition, the spleens of mice administered with FOLFOX chemotherapy regimen were significantly enlarged, while the spleens of the mice in the Erianin + FOLFOX combination group were significantly reduced (Figure 4B), and the results of calculating the organ index of liver was also consistent (Figure 4C). The results show that Erianin can improve the abnormal appearance of mice caused by FOLFOX chemotherapy regimen to a certain extent.

### 3.5 Erianin significantly attenuates FOLFOX-induced organ injury

**FIGURE 5:**
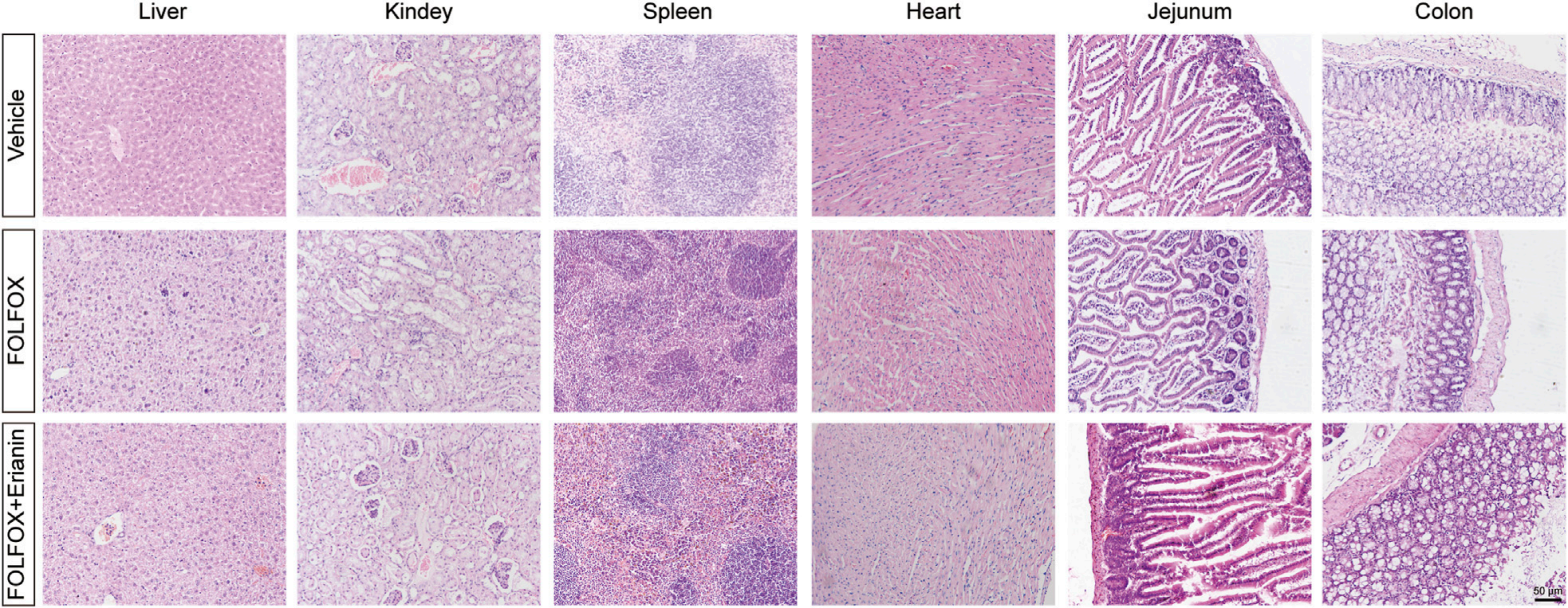
Histopathological examination by H&E staining in liver, kidney, spleen, heart, jejunum and colon tissues (Scale bar: 50 μm).

While FOLFOX demonstrates potent antitumor efficacy, its dose-limiting toxicity remains clinically significant. Gastrointestinal tract injury and immune organ toxicity constitute primary adverse effects. The mice in FOLFOX group shows that their intestinal villi atrophying, lamina propria thickening, intestinal crypt abnormal deepening and area of white pulp in spleen decreasing. These findings confirm substantial FOLFOX-associated toxicity. Crucially, Erianin co-administration significantly mitigated both intestine and spleen pathological alterations (Figure 5). These protective effect against FOLFOX-induced organ injury demonstrates Erianin’s potential as a chemoprotective adjuvant.

### 3.6 Erianin alleviates FOLFOX-induced hematological toxicity

**FIGURE 6:**
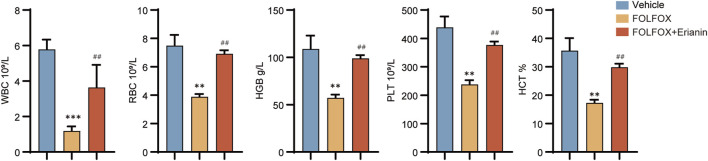
Protective effects of Erianin on peripheral blood parameters in FOLFOX-treated mice (WBC, RBC, HGB, PLT, HCT) (n = 5). Data were presented as mean ± SEM. **p < 0.01, ***p < 0.001 vs. vehicle; ^*##*^ *p < 0.01* vs. FOLFOX monotherapy.

To evaluate Erianin’s protective effects against FOLFOX-induced bone marrow suppression, we collected and analyzed complete blood count (CBC) parameters from murine peripheral blood. Compared to vehicle, FOLFOX monotherapy significantly reduced white blood cells (WBC), red blood cells (RBC), hemoglobin (HGB), platelets (PLT), and Hematocrit (HCT). Notably, Erianin + FOLFOX combination significantly attenuated these cytopenias (Figure 6). These findings suggest that Erianin’s capacity to mitigate FOLFOX-induced hematopoietic toxicity.

### 3.7 Erianin ameliorates FOLFOX-induced intestinal barrier dysfunction

**FIGURE 7:**
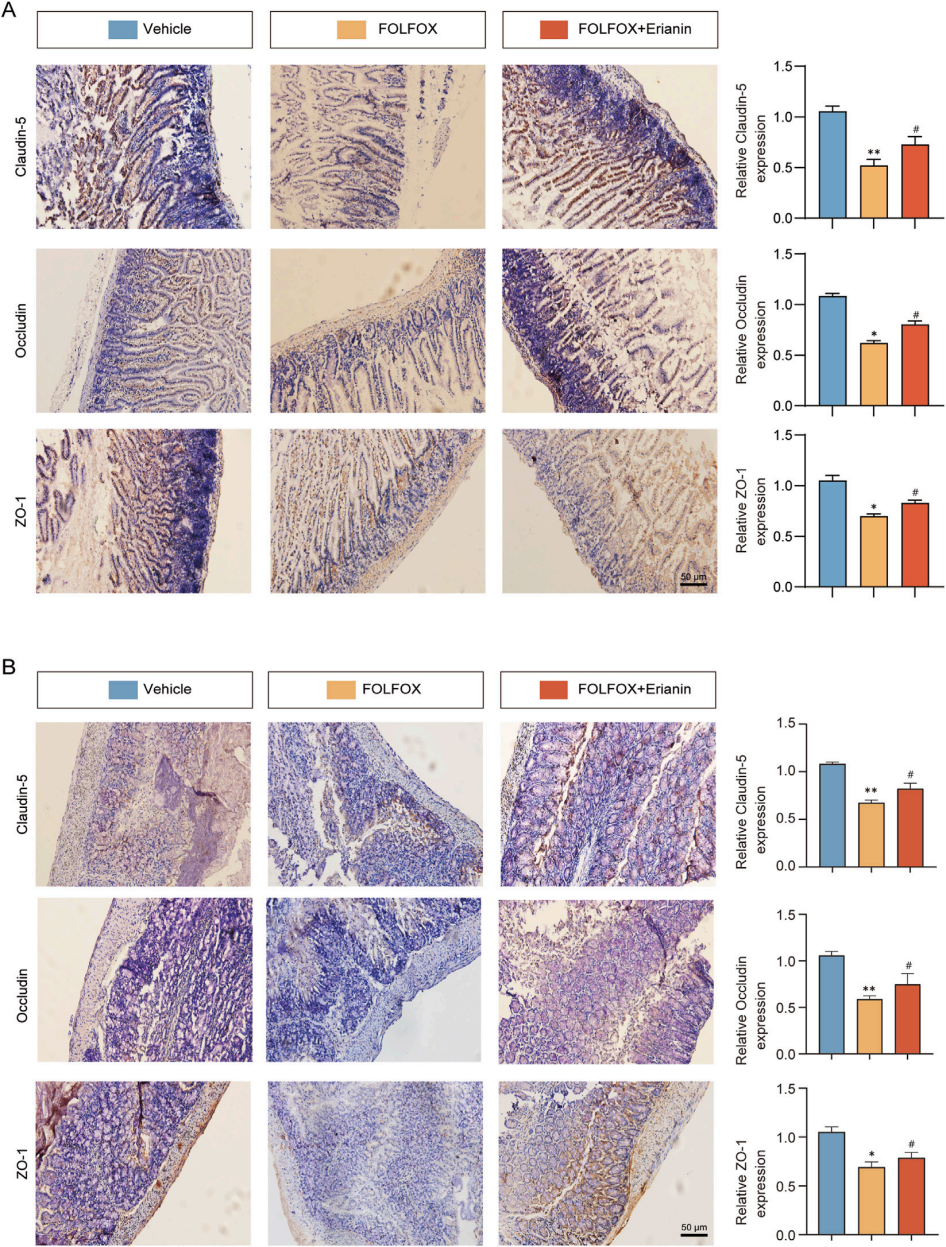
Erianin alleviates FOLFOX-induced intestinal barrier damage by preserving tight junction proteins. **(A)** IHC results of ZO-1, Occludin and Claudin-5 in jejunum (Left: Representative images. Right: Quantitative analysis). **(B)** Corresponding protein detection in colon tissues (Scale bar: 50 μm). Data were presented as mean ± SEM. *p < 0.05, **p < 0.01 vs. vehicle; ^*#*^ *p < 0.05* vs. FOLFOX monotherapy.

FOLFOX chemotherapy downregulates key tight junction proteins, such as ZO-1, Occludin, and Claudin-5, compromising intestinal mucosal barrier integrity. IHC analysis revealed that compared to FOLFOX monotherapy, Erianin co-administration significantly upregulated expression of these critical barrier proteins (Figures 7A,B). These findings suggest that Erianin + FOLFOX combination therapy effectively restores intestinal barrier function.

### 3.8 Erianin attenuates FOLFOX-induced intestinal damage by modulating the AKT1/mTOR signaling pathway

**FIGURE 8:**
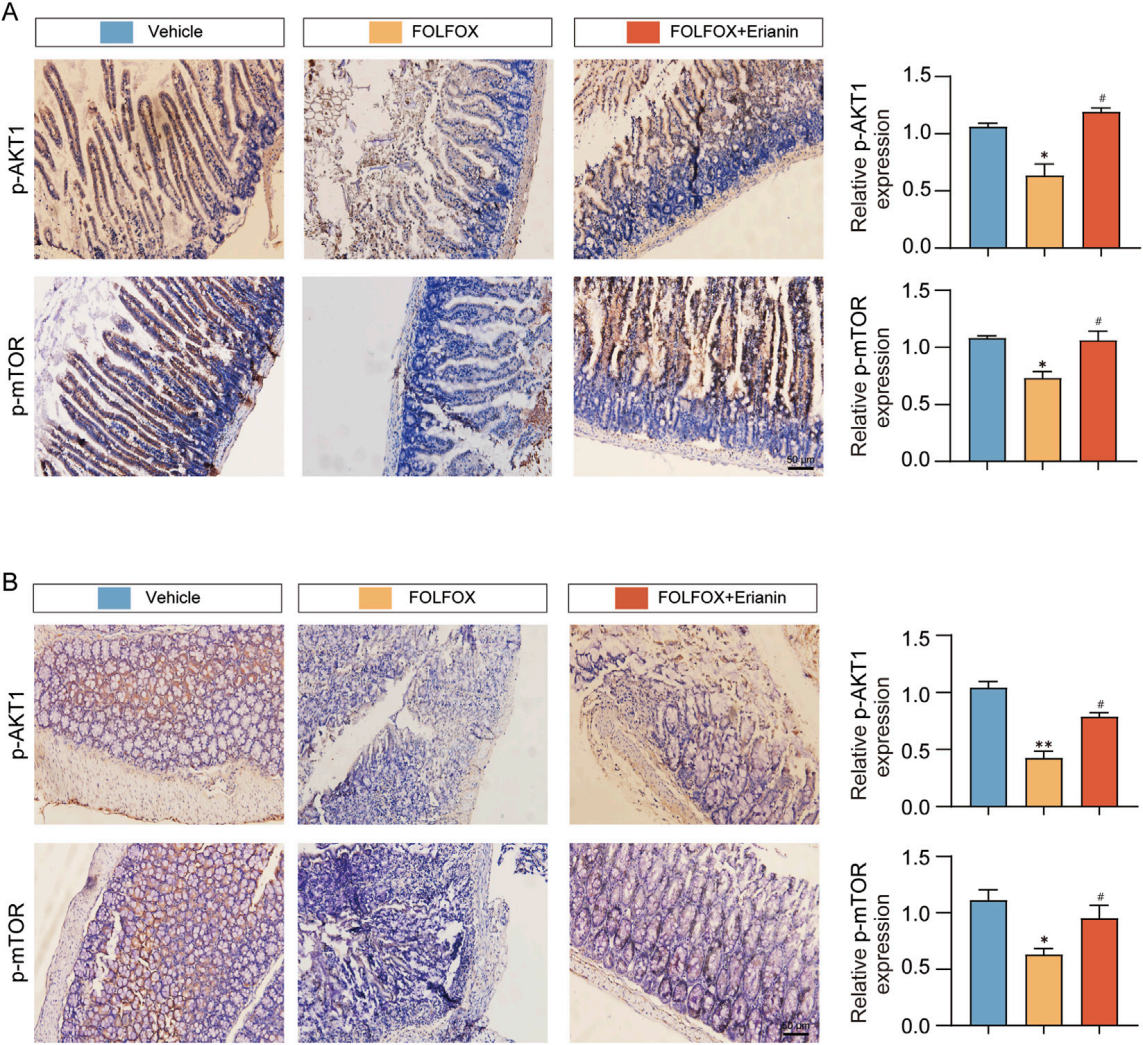
Erianin restores intestinal barrier via AKT1/mTOR activation. **(A)** Erianin antagonizes FOLFOX-induced inhibition of AKT1/mTOR phosphorylation in jejunum. **(B)** Identical regulatory pattern observed in colonic tissues by IHC (Scale bar: 50 μm). Data were presented as mean ± SEM. *p < 0.05, **p < 0.01 vs. vehicle; ^*#*^ *p < 0.05* vs. FOLFOX monotherapy.

Molecular docking analyses showed that Erianin binds with high affinity to AKT1. IHC results showed that the phosphorylation levels of p-AKT1 and p-mTOR were significantly reduced in the small intestinal villi and colon tissues in the FOLFOX group compared with the vehicle group, and the expression was restored by the combination treatment of Erianin (Figures 8A,B). These results suggest that Erianin may antagonise FOLFOX chemotherapy-induced intestinal damage by activating the AKT1/mTOR signal pathway and regulating intestinal epithelial cell repair.

### 3.9 Erianin enhances the anti-tumor effect of FOLFOX in CRC

**FIGURE 9:**
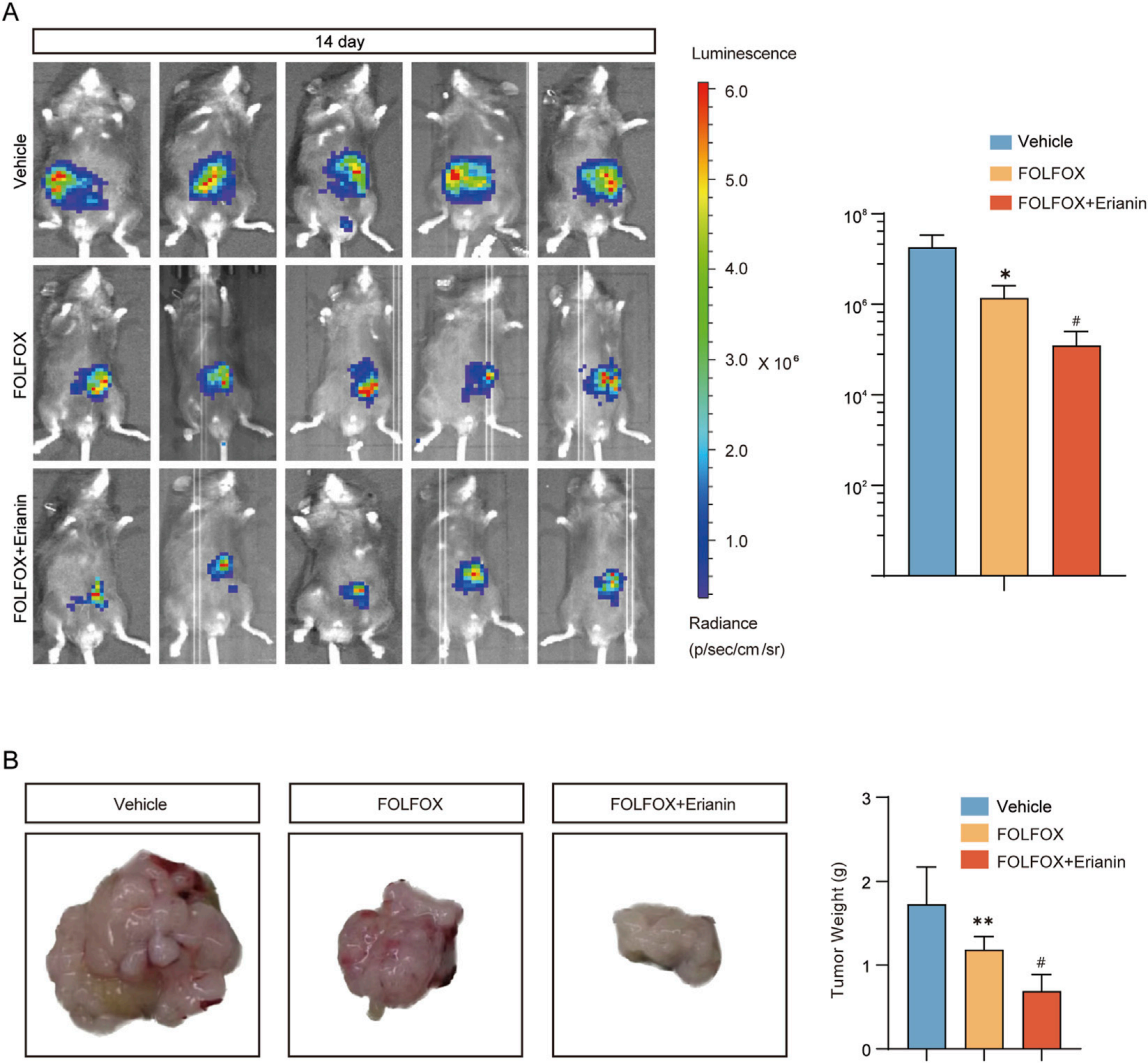
Synergistic inhibition of CRC growth by Erianin and FOLFOX. **(A)** *In vivo* imaging system monitoring tumor growth kinetics (Left) and fluorescence quantification (Right). **(B)** Tumor specimens (Left) with weight statistical analysis (Right). n = 5. Data were presented as mean ± SEM. *p < 0.05,**p < 0.01 vs. vehicle; ^*#*^ *p < 0.05* vs. FOLFOX monotherapy.

Using an MC38 mouse CRC orthotopic model, we investigated the enhanced antitumor efficacy of Erianin combined with FOLFOX chemotherapy. Compared with vehicle group, FOLFOX monotherapy significantly inhibited tumor growth (Figure 9A) and reduced tumor weight (Figure 9B). Notably, the Erianin + FOLFOX combination group exhibited further reduction in both tumor volumes and weight when compared with FOLFOX alone, indicating that Erianin can enhance FOLFOX-mediated anti-tumor effects.

### 3.10 Erianin enhances the ability of FOLFOX to inhibit tumor cell proliferation and promote apoptosis

**FIGURE 10:**
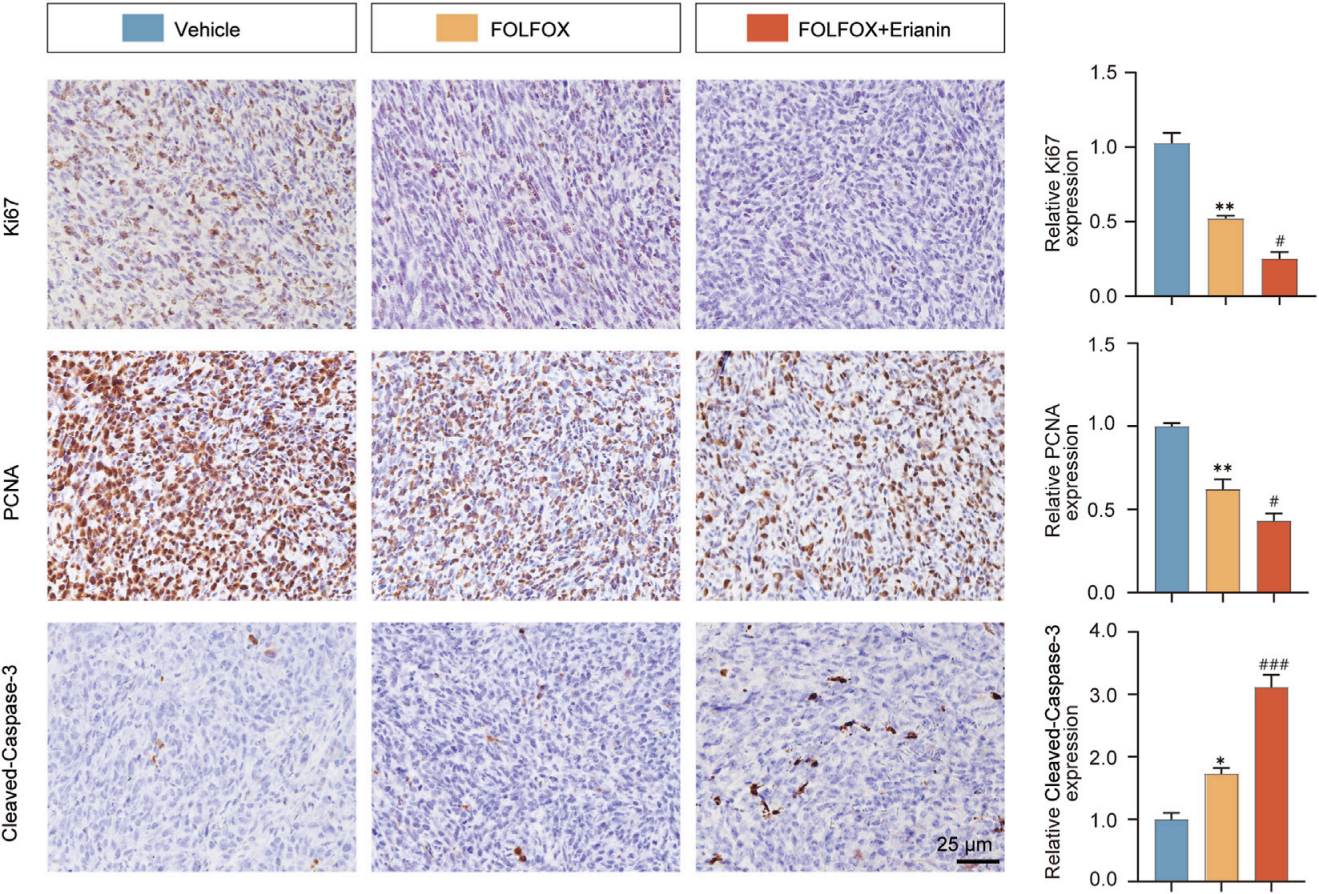
Erianin synergizes with FOLFOX to modulate tumor apoptosis and proliferation. Left: IHC staining of Ki67, PCNA (proliferation) and Cleaved Caspase-3 (apoptosis). Right: Quantitative analysis. Scale bar: 25 μm. Data were presented as mean ± SEM. *p < 0.05, **p < 0.01 vs. vehicle, ^*#*^ *p < 0.05,* ^*###*^ *p < 0.001* vs. FOLFOX monotherapy.

Using IHC, we evaluated how Erianin combined with FOLFOX inhibits proliferation and induce apoptosis in CRC cells. Compared to FOLFOX monotherapy, Erianin + FOLFOX combination group significantly enhanced suppression of proliferation biomarkers Ki67 and PCNA. Concurrently, Erianin + FOLFOX combination group markedly upregulated Cleaved Caspase-3 expression, a key executioner of apoptosis (Figure 10). The findings indicate that Erianin potentiates FOLFOX-mediated inhibition of proliferation and induction of apoptosis.

## 4 Discussion

CRC is the third most common malignant tumor globally, with chemotherapy serving as the primary treatment for advanced--stage patients. Currently, the FOLFOX chemotherapy, while established as a first-line standard, is limited by significant toxicity, drug resistance and serious treatment-related complications (Andreyev et al., 2014; Gibson and Keefe, 2006; Mohelnikova-Duchonova et al., 2014). Therefore, discovering agents that enhance FOLFOX’s efficacy while mitigating its toxicity represents an urgent therapeutic imperative to improve CRC treatment outcomes.

TCM demonstrates unique advantages in oncology through reducing chemotherapy adverse effects, enhancing chemosensitivity, and promoting quality of life (Wang et al., 2020; Zhang et al., 2020). *Dendrobium chrysotoxum* Lindl, a typical medicinal-food homologous material, has gained significant attention in anti-tumor drug development. Its primary active ingredient, Erianin (a bibenzyl compound), was investigated herein. Previous data showed that Erianin has a broad-spectrum anti-tumor effect, including regulating signaling pathways such as PI3K/AKT (Xu et al., 2021; Zhang et al., 2021), REST/LRSAM1 (Mansuer et al., 2024), NRF2 (Xiang et al., 2021), and MEK1/2 (Zhu et al., 2025), etc. These mechanisms inhibit the proliferation across multiple cancer types, such as lung, colon, breast, and pancreatic cancer, and prevent the occurrence and progress of tumors. Besides, Erianin synergizes with 5-fluorouracil to inhibit the LKB1-SIK2/3-PARD3 pathway in gastric cancer, reversing the expression of EMT-related proteins (Wei et al., 2024). Erianin also inhibits the metastasis in A549 and cisplatin-resistant A549/DDP lung cancer cells, overcoming cisplatin resistance (Tang et al., 2024). In our study, the combined treatment group showed a superior tumor growth suppression versus stronger anti-CRC growth effect than the FOLFOX monotherapy, with significant reduction in tumor volume and weight. The IHC results showed that Erianin can further reduce the proportion of Ki67 and PCNA positive cancer cells and increase the content of the tumor apoptosis marker Cleaved Caspase-3. Although our experimental results suggest that Erianin can enhance the antitumor efficacy of FOLFOX *in vivo*, the specific enhanced mechanism has not been explored in depth. Based on the existing literature, oxaliplatin primarily induces DNA damage by forming both intra-strand and inter-strand crosslinks at the N7 position of adenine (A) and guanine (G) bases (Osmanoğulları et al., 2023). 5-Fluorouracil (5-FU) induces DNA damage by inhibiting thymidylate synthase (TS) and incorporating its metabolites (FUTP/FdUTP) into RNA/DNA, thereby disrupting tumor cell DNA synthesis (Averill et al., 2024). DNA-damaging effects based on 5-fluorouracil and oxaliplatin, and pro-apoptotic properties of Erianin, we propose a novel mechanistic hypothesis that Erianin enhances the anticancer efficacy of FOLFOX by inhibiting the nucleotide excision repair (NER) pathway, thereby exacerbating DNA damage and generating enhanced lethality of FOLFOX chemotherapy (Balin-Gauthier et al., 2008). We will explore this hypothesis more deeply in the future.

FOLFOX has serious toxic side effects in clinical practice, mainly reflected in acute and neurological lesions, manifested as numbness in the hands and feet, and persistent neuropathy; gastrointestinal side effects are manifested as nausea and vomiting, decreased appetite, indigestion, etc. (Zhang et al., 2022); in addition, FOLFOX will also affect the hematopoietic system, manifested as anemia, a decrease in the number of white blood cells, granulocytes and platelets; and affect the patient’s liver and renal function (Li et al., 2011; Mahadevappa et al., 2024). Erianin was used at 10 mg/kg, a dose that has been confirmed to be safe in our previous colorectal and pancreatic cancer model studies (within the 50–100 mg/kg safe dose range) (Miao et al., 2023; Zhu et al., 2025). The dose conversion using body surface area normalization revealed that the mouse FOLFOX regimen (5-fluorouracil 50 mg kg^-1^, oxaliplatin 10 mg kg^-1^ and calcium folinate 90 mg kg^-1^) correspond to human equivalents of 2,000 mg/m^2^ 5-fluorouracil, 130 mg/m^2^ oxaliplatin, and 400 mg/m^2^ leucovorin. This dosage is clinically consistent with the norms of use (de Gramont et al., 2003; Mei et al., 2023). Meanwhile, these dose equivalents are consistently associated with grade 3–4 adverse events, particularly neutropenia and vomiting-related gastrointestinal toxicity, in human patients (Folprecht et al., 2014; Wasan et al., 2017). In a study of 667 patients with stage III (CRC), all patients were treated with FOLFOX adjuvant chemotherapy regimen. The results showed that 54% of the patients experienced neutropenia, 33.1% experienced anemia, and 36.9% experienced severe vomiting (Uncu et al., 2013).

Although available adjuvant medications (such as amifostine and dexamethasone) can partially mitigate chemotherapy-induced toxicities, certain unacceptable side effects persist. For instance, amifostine may induce hypotension, while dexamethasone can lead to metabolic disturbances. These limitations can also add to the burden of patient care in the clinical setting (Hayashi et al., 2021; Koukourakis et al., 2009). In contrast, Erianin exhibits significant advantages, it has higher safety with little organ toxicity, making it suitable for long-term adjuvant use. And it has stronger targeting, directly acting on the mechanisms of gastrointestinal damage caused by FOLFOX. Furthermore, it can achieve enhanced effects by protecting normal tissues while enhancing the tumor-killing effect of chemotherapy. This property makes Erianin more suitable than traditional adjuvants for the clinical needs of the FOLFOX regimen.

AKT/mTOR signaling plays a pivotal role in intestinal barrier repair by upregulating tight junction proteins (ZO-1/Occludin), inhibiting epithelial apoptosis, and promoting stem cell proliferation. Huangqin decoction was shown to ameliorate intestinal barrier dysfunction by activating the mTOR signaling pathway via upregulation of amino acid metabolism (Li et al., 2022). Similarly, Paeoniflorin significantly alleviated dextran sulfate sodium -induced colitis by promoting intestinal stem cell renewal and differentiation through PI3K-AKT-mTOR pathway regulation (Ma et al., 2023). In this study, we integrated network toxicology and molecular docking techniques to confirm that Erianin binds to AKT1 with high affinity (binding energy −6.4 kcal/mol). *In vivo* experiments showed that Erianin significantly repaired intestinal barrier function by activating the AKT1/mTOR pathway. These changes were consistent with phenotypic improvement, and the combination group restored abnormal body weight and appearance, ameliorated splenic and colonic damage, and alleviated bone marrow suppression in FOLFOX group mice. While we have focused on gastrointestinal and hematologic toxicity caused by the FOLFOX regimen in the current study, it is important to note that the range of chemotherapy-related adverse reactions is much broader, especially neurotoxicity, a major challenge in clinical care. Oxaliplatin-induced peripheral neuropathy (OIPN) presents with symptoms such as sensory abnormalities and nociceptive sensitization, which severely affects patients’ quality of life and often leads to treatment interruption (Stefansson and Nygren, 2016). In the future, we will systematically evaluate the effects of Erianin on OIPN and other toxicities, including the establishment of animal models (e.g., patient-derived xenograft models) of neuropathy, the detection of changes in nerve conduction velocity, and the analysis of the expression of relevant molecular markers, in order to provide a more comprehensive experimental basis for its clinical translation.

## 5 Conclusion

In summary, we employed toxicological network pharmacology combined with molecular docking and orthotopic MC38 CRC model to evaluate the alleviation of FOLFOX-induced gastrointestinal toxicity by Erianin, a bibenzyl compound derived from *D. chrysotoxum* Lindl. This work is expected to develop Erianin as a potential chemotherapy-assisted therapy in the clinic, increasing efficacy while reducing toxic side effects. Our findings position Erianin as a novel chemoadjuvant candidate for clinical translation.

## Data Availability

The original contributions presented in the study are included in the article/Supplementary Material, further inquiries can be directed to the corresponding authors.
