# Supplementary material for: Natural product Erianin: mitigating FOLFOX toxicity and enhancing against colorectal cancer
Source: Front Chem. 2025 Aug 22;13:1650197. doi: 10.3389/fchem.2025.1650197 (PMC12411860; doi:10.3389/fchem.2025.1650197)
Supplement: Supplementary file 1 [file DataSheet1.pdf]

辰光生物 Herbest

Baoji Herbest Bio-Tech Co., Ltd

Tel：0086-917- 8883868
Fax：0086-917- 3139802
Email: info@herbest.cn
Web: http://www.herbest.cn

Certificate of Analysis

产品名称 **Product Name**	毛兰素 Erianin
Cas No:	95041-90-0
批号 Batch No	HR2318W9
分子量 Mol.Weight	318.4g/mol
分子式 Mol.Formula	$C_{18}H_{22}O_5$

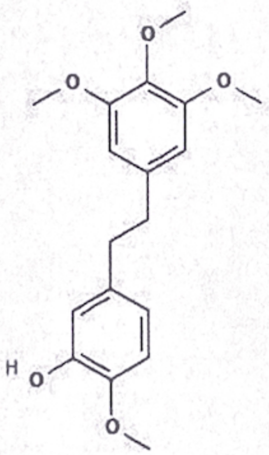

**项目 Items**	标 准 Specifications	结 果 Analysis Results
**性状 Appearance**	白色粉末 White powder	符合 Confirms
**Assay by HPLC**	≥ 98%	99.311%
**结论 Conclusion**	符合企业标准<br>Complies with the Enterprise Standard	
**贮存 Storage**	2~4℃密封保存，置阴凉干燥处.<br>2~4℃store in a cool and dry area,sealed and keep from direct light	
**有效期 Expiration**	2年 2 years	

Herbest 辰光生物
Baoji Herbest Bio-Tech Co., Ltd

Tel：0086-917- 8883868
Fax：0086-917- 3139802
Email: info@herbest.cn
Web: http://www.herbest.cn

HPLC:

检测方法（Method）	色谱柱（Column）：Welch C18 (4.6*250mm，5um)<br>流动相（Mobile phase）： A 甲醇：B 水 0-15min 70%A：15-30min 70%-95% A<br>UV 检测器（Derector）：250nm<br>柱温：30℃<br>样品浓度：1.4mg/ml(Methanol)<br>进样量：20ul

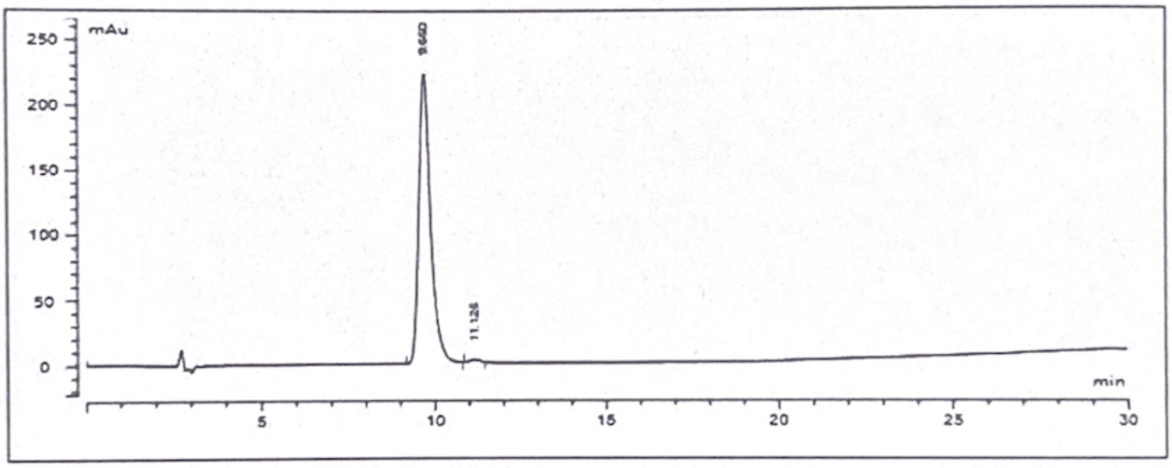

No	名称	保留时间(min)	峰面积(mAu*s)	峰宽(min)	半峰宽(min)	峰高(mAu)	面积百分比(%)	峰类型
1	N.A.	9.650	5299.36038	0.669	0.394	224.281	99.311	BB
2	N.A.	11.125	36.76195	0.539	0.317	1.933	0.689	BB
3	总计		5336.12233				100.000	

质检专用章
